# Formation of Lung Inducible Bronchus Associated Lymphoid Tissue Is Regulated by *Mycobacterium tuberculosis* Expressed Determinants

**DOI:** 10.3389/fimmu.2020.01325

**Published:** 2020-06-30

**Authors:** Micah D. Dunlap, Oliver A. Prince, Javier Rangel-Moreno, Kimberly A. Thomas, Julia M. Scordo, Jordi B. Torrelles, Jeffery Cox, Adrie J. C. Steyn, Joaquín Zúñiga, Deepak Kaushal, Shabaana A. Khader

**Affiliations:** ^1^Department of Pathology and Immunology, Washington University School of Medicine, St. Louis, MO, United States; ^2^Department of Molecular Microbiology, Washington University School of Medicine, St. Louis, MO, United States; ^3^University of Rochester Medical Center, Rochester, NY, United States; ^4^Texas Biomedical Research Institute, San Antonio, TX, United States; ^5^Department of Molecular and Cell Biology, University of California, Berkeley, Berkeley, CA, United States; ^6^Department of Microbiology, Centers for AIDS Research and Free Radical Biology, University of Alabama at Alabama, Birmingham, AL, United States; ^7^African Health Research Institute (AHRI), Durban, South Africa; ^8^Instituto Nacional de Enfermedades Respiratorias, Mexico City, Mexico; ^9^Division of Bacteriology, Tulane National Primate Research Center, Covington, LA, United States; ^10^Department of Microbiology and Immunology, Tulane University School of Medicine, New Orleans, LA, United States

**Keywords:** Tuberculosis, lung, innate immunity, pulmonary, epithelial cells, iBALT

## Abstract

*Mycobacterium tuberculosis* (*Mtb*) is the causative agent of the infectious disease tuberculosis (TB), which is a leading cause of death worldwide. Approximately one fourth of the world's population is infected with *Mtb*. A major unresolved question is delineating the inducers of protective long-lasting immune response without inducing overt, lung inflammation. Previous studies have shown that the presence of inducible Bronchus-Associated Lymphoid Tissue (iBALT) correlate with protection from *Mtb* infection. In this study, we hypothesized that specific *Mtb* factors could influence the formation of iBALT, thus skewing the outcome of TB disease. We infected non-human primates (NHPs) with a transposon mutant library of *Mtb*, and identified specific *Mtb* mutants that were over-represented within iBALT-containing granulomas. A major pathway reflected in these mutants was *Mtb* cell wall lipid transport and metabolism. We mechanistically addressed the function of one such *Mtb* mutant lacking mycobacteria membrane protein large 7 (*MmpL7*), which transports phthiocerol dimycocerosate (PDIM) to the mycobacterial outer membrane (MOM). Accordingly, murine aerosol infection with the *Mtb* mutant Δ*mmpl7* correlated with increased iBALT-containing granulomas. Our studies showed that the Δ*mmpl7* mutant lacking PDIMs on the surface overexpressed diacyl trehaloses (DATs) in the cell wall, which altered the cytokine/chemokine production of epithelial and myeloid cells, thus leading to a dampened inflammatory response. Thus, this study describes an *Mtb* specific factor that participates in the induction of iBALT formation during TB by directly modulating cytokine and chemokine production in host cells.

## Introduction

*Mycobacterium tuberculosis* (*Mtb*) is the causative agent of pulmonary tuberculosis (TB). TB is caused by inhalation of *Mtb* bacilli and it is currently estimated that approximately one fourth of the world's population is infected with *Mtb* (1). While most individuals infected with *Mtb* are asymptomatic and do not exhibit clinical symptoms, 5–10% of infected individuals can progress to pulmonary clinical TB. The immune parameters that distinguish latent TB from active pulmonary TB are not as yet clearly defined.

The first interactions between *Mtb* and the host happen in the lung airways immediately after inhalation of *Mtb* bacilli. These early interactions involve alveolar macrophages (2) (AMs), dendritic cells (3, 4) (DCs), and epithelial cells lining the airway (5–7) and have the potential to drive immune responses. As a result of initiation of immune responses, the tubercle granuloma is formed, which is a hallmark immune structure formed during TB (8). We have previously shown that protective granulomas that are formed during latent TB are associated with the formation of B-cell containing lymphoid follicles (9). During severe active TB, granulomas that do not effectively contain *Mtb* are comprised predominately of neutrophils (10, 11) and permissive monocytes (12), which have been implicated in general tissue destruction, thus skewing responses toward disease progression. Non-protective granulomas are hallmarked by the formation of hypoxic, necrotic cores that do not prevent *Mtb* growth and eventually lead to dissemination to other organs and tissues (10). The earliest mechanism(s) which determine the nature and outcome of granulomas during infection remains elusive, and are a focus of this work.

In this study, we aimed to determine the *Mtb* and host specific factors that drive the formation of inducible bronchus associated lymphoid tissue (iBALT) during TB. To examine the *Mtb* specific factors involved, we utilized an *Mtb* transposon mutant library to screen the induction of lymphoid follicles using the Non-Human primate (NHP) model of pulmonary TB. This screen allowed us to identify *Mtb* genes which when mutated led to increased induction of iBALT within granulomas. The NHP model exhibits characteristics associated with human TB including the pulmonary cellular and acellular lesions (13, 14). We found an over-representation of *Mtb* cell wall mutants within iBALT containing granulomas and further characterized the role of one such mutant using the mouse model of TB. The mycobacteria membrane protein large 7 (*Mmpl7*) inner membrane protein is best known for the transport of phthiocerol dimycocerosate (PDIM) to the free fatty acid layer of the mycobacterial outer membrane (MOM) (15, 16). The role of lipid presentation in the MOM is well-documented in modulating various host responses (17–19), suggesting that MOM lipids contact with host cells in the lung plays a role in disease progression. In light of these observations, we aimed to understand how the *Mtb* Δ*mmpl7* cell wall mutant identified in our screen modulates early epithelial signaling and myeloid recruitment in order to coordinate granuloma structure and the formation of iBALT. In this study we found that in the mouse model, infection with the Δ*mmpl7* mutant drives decreased bacterial burden and increased formation of iBALT, as was observed in NHPs. Furthermore, the Δ*mmpl7* mutant also drove decreased inflammatory cytokine and chemokine production *in vitro* and *in vivo*, along with decreased accumulation of myeloid and lymphoid immune cells. We demonstrate that the Δ*mmpl7* mutant overexpresses diacyl trehaloses (DATs), a *Mtb* cell wall lipid, which can drive the observed decreased inflammatory cytokines and chemokine production by *in vitro* macrophages and also yields increased production of IL-10.

## Results

### Identification of *Mtb* Genes Associated With Formation of Protective Lymphoid Follicles

**Figure 1:**
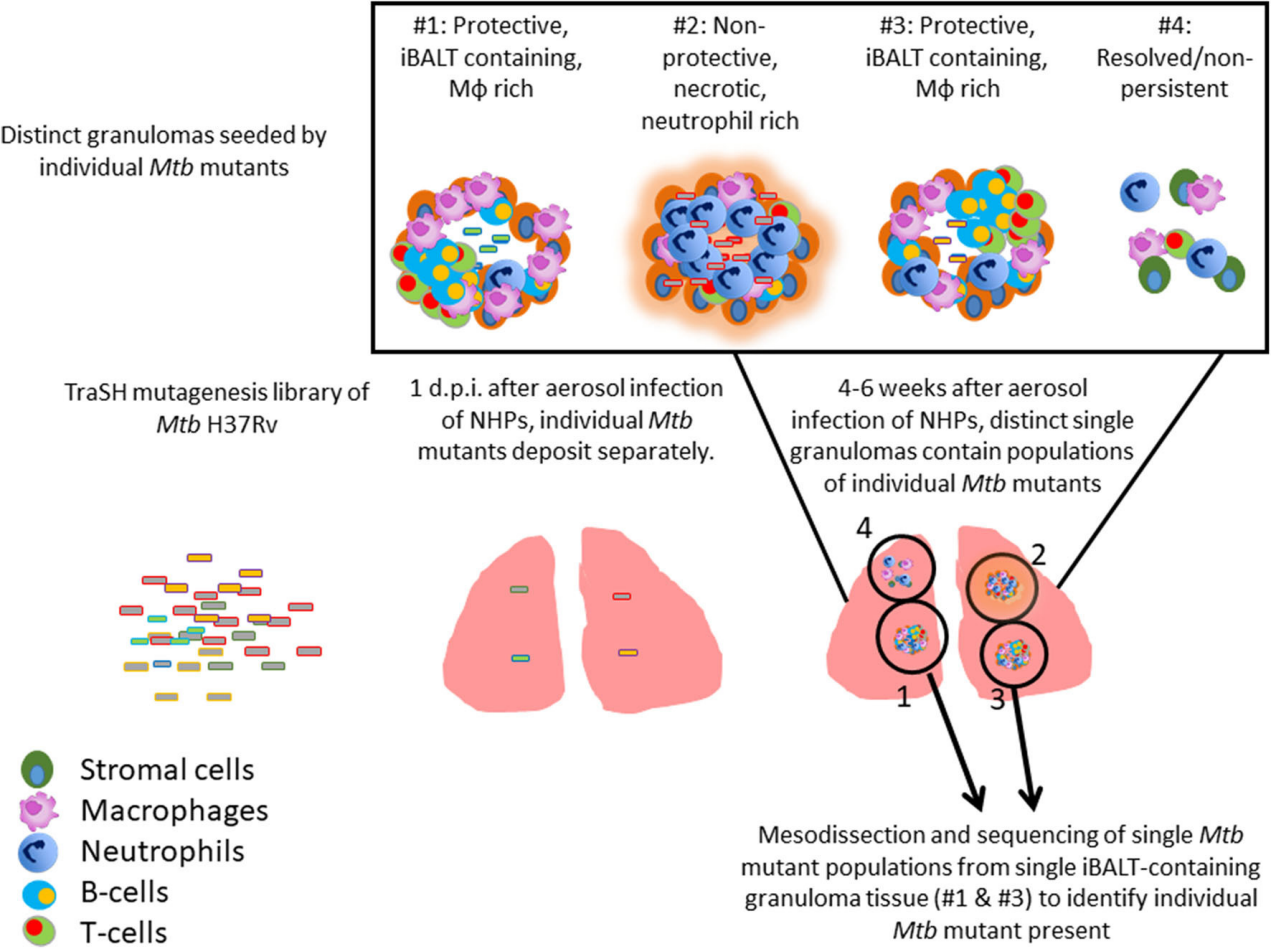
Identification of Mtb genes associated with formation of protective lymphoid follicles: Transposon site hybridization library (TraSH) using the himar1 transposon mutagenesis strategy was employed to create a *Mtb* mutant library for the specific discovery of *Mtb* genes influencing the progression of lymphoid follicle containing granulomas in NHPs. NHPs were infected with 100,000 CFU of *Mtb* mutants and lung sections were harvested 4–6 weeks after infection. In combination with mesodissection, the analysis of >1,200 separate lesions identified nine *Mtb* single mutants that were highly represented within protective iBALT containing TB granulomas in macaques. Single *Mtb* mutants were isolated from single granulomas.

TB granulomas contain distinct iBALT structures which are protective in mice and macaques (9). NHPs infected with *Mtb* exhibit the spectrum of disease severity observed clinically during human TB, with a diverse array of granuloma structures reflected by differences in immune cell recruitment and disease outcome (14, 20). Thus, we used the NHP model to probe the early host-*Mtb* interactions that mediate the signaling events that initiate the induction of iBALT within TB granulomas. NHPs were infected with 1 × 10^5^ CFU of *Mtb* (H37Rv) mutants from the himar1 transposon mutagenesis site hybridization (TraSH) library (21, 22). Four-six weeks post-infection, the animals were humanely euthanized due to TB disease. At this time, macaque lungs demonstrated a wide distribution of caseous and follicular granulomatous structures (Figure 1), with follicular granulomas featuring prominent cellular structure. In combination with mesodissection, DNA sequencing analysis of separate B cell follicle containing granulomas, seeded by *Mtb* mutants (23, 24), identified nine *Mtb* single mutants that were highly represented within distinct iBALT containing TB granulomas in macaques (Figure 1 and Table 1). Of the *Mtb* mutants represented within these “protective” granulomas, two members of the mycobacterial membrane protein large family (*Mmpl2* and *Mmpl7*), were well-reflected. *MmpL2* is a putative inner membrane transporter protein with unknown substrate, while *MmpL7* is an inner membrane protein associated with phthiocerol dimycocerosate (PDIM) transport to the MOM (15, 16). The NAD dehydrogenase, *NdhA* is a non-essential protein found in the inner mycobacterial membrane associated with NADH mediated electron transfer to the electron transport chain (25). *EccD5* is an essential inner membrane protein that makes up part of the type VII secretion system, the absence of which results in sensitivity to detergents and killing by macrophage (26). *ClpB* and *Acr2* are both heat stress molecular chaperones regulated by the alternative sigma factors *SigH* and *SigE*, respectively (27). Of the two, only *ClpB* is essential for *in vitro* growth. The non-redundant glutamine synthetases *GlnA2* and *GlnA4* are involved in nitrogen metabolism but are not associated with any loss of virulence *in vivo* (28). *Cmtr* is a cytosolic sensor of cadmium Cd(II) and lead Pb(II) (29) which are trace metal contaminants found in the lung (Table 1).

**Table 1:** Identification of Mtb genes associated with formation of protective lymphoid follicles: Transposon site hybridization library (TraSH) using the himar1 transposon mutagenesis strategy was employed to create a *Mtb* mutant library for the specific discovery of *Mtb* genes influencing the progression of lymphoid follicle containing granulomas in NHPs.

***Mtb* genes identified**	**Putative function**
**from NHP transposon screen**	
*mmpL2* (Rv0507) 106 kDa	Fatty acid transport
*mmpL7* (Rv2942) 95 kDa	Fatty acid transport
*ndhA* (Rv0392c) 50 kDa	Electron transport
*eccD5* (Rv1795) 53 kDa	Type VII secretion (^*^)
*clpB* (Rv0384c)	Molecular chaperone (^*^)
*acr2* (Rv0251c)	Molecular chaperone
*glnA2* (Rv2222c)	Glutamine biosynthesis
*glnA4* (Rv2860c)	Glutamine biosynthesis
*cmtR* (Rv1994c)	Metal sensor, transcriptional regulator

NHPs were infected with 1 × 10^5^ CFU of Mtb H37Rv mutants and lung sections were harvested 4–6 weeks after infection. In combination with mesodissection, the analysis of >1,200 lesions identified 9 Mtb mutants that were highly represented within protective iBALT containing TB granulomas in macaques.

* *Essential gene*.

Taken together, the *Mtb* transposon screen conducted in NHPs has identified a novel set of *Mtb* genes involved in generation of lymphoid follciles within the lung, that appear to control *Mtb* metabolism and inner membrane lipid transport. In this study, we chose to mainly focus on the interaction driven by *Mtb Mmpl7* that mediates formation of the granuloma.

### Δ*mmpl*7 Mutant Drives *Mtb* Enhanced B Cell Follicle Containing iBALT Formation in Mouse Model

**Figure 2:**
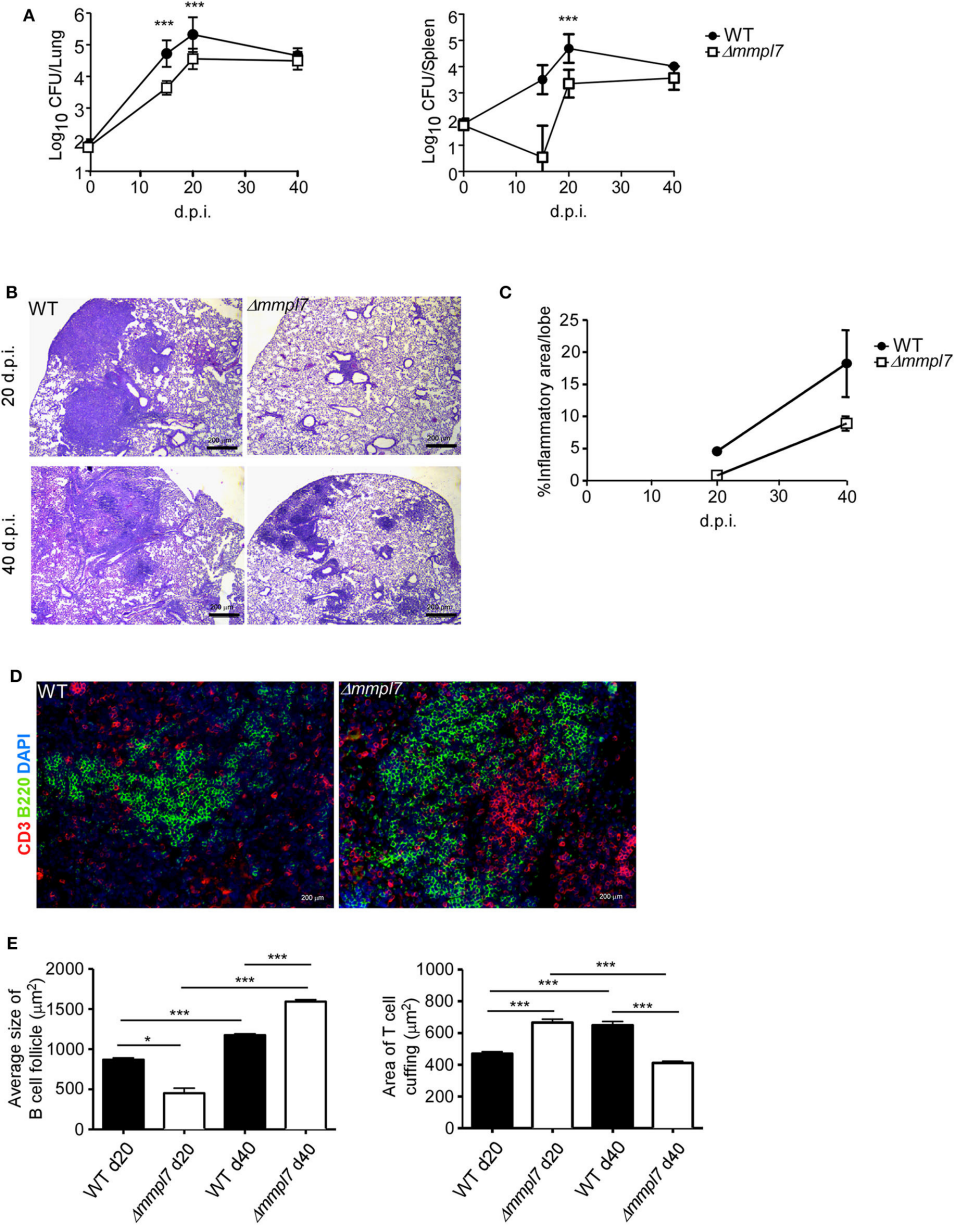
Δmmpl7 mutant drives Mtb enhanced B cell follicle formation in mouse model: Sex and age matched C57BL/6 mice were infected with 100 CFU of *Mtb* Erdman WT or Δ*mmpl7*. **(A)** Lung and spleen homogenates were used to determine *Mtb* CFU counts at indicated time points. **(B,C)** Lungs from *Mtb*-infected mice at 20 (top) and 40 (bottom) d.p.i. were formalin fixed, embedded in paraffin and used for H&E staining and inflammation was quantified by tracing areas. **(D,E)** B cell follicles present within lung sections were visualized by confocal microscopy. Slides were visualized and quantified by outlining the lesions using the automated tool of the Zeiss Axioplan 2 microscope. Infected groups (*n* ≥ 5) comparing WT Erdman and Δ*mmpl7 Mtb* strains at individual time points were compared using Student's *t*-test. Mean and standard deviation (SD) were plotted for each group at each indicated time point. *p < 0.05, **p < 0.01, ***p < 0.001.

To determine the functional role of *Mtb Mmpl7* in modulating iBALT generation *in vivo*, we infected C57BL/6 mice (B6) with 100 CFU *Mtb* Erdman or the Δ*mmpl7* mutant Erdman strain and characterized immune recruitment and induction of iBALT features in the lung. As previously shown, infection with the Δ*mmpl7* mutant resulted in early decreased *Mtb* CFU which recovered by 40 days post-infection (d.p.i.) in the lung and spleen (Figure 2A). At a time when *Mtb* CFU was comparable between the WT and mutant strains, we observed that the total inflammation observed in the lungs of the Δ*mmpl7* mutant infected mice was significantly decreased (Figures 2B,C). However, despite significantly decreased overall inflammation, infection with the Δ*mmpl7* mutant resulted in increased formation of B cell lymphoid follicle containing area, reflecting iBALT areas (Figures 2D–E). Furthermore, by 40 d.p.i., T cells were well able to infiltrate within iBALT containing granulomas, as the area of T cell cuffing around the granulomas was decreased in Δ*mmpl7* mutant infected mice (Figures 2D,E). Together, these findings suggest that *Mtb* lacking the *Mmpl7* gene induces improved induction of iBALT and decreased inflammation, suggesting that *Mtb* actively shuts down iBALT formation utilizing virulence factors such as PDIM, or alternatively, the reduced early *Mtb* growth in the *Mmpl7* mutant *in vivo* propagates iBALT containing granulomas.

It has been shown previously that the Δ*mmpl7* mutant lacks the ability to transport PDIMs, an important *Mtb* structural lipid and virulence factor, to the MOM, leading to an early growth defect *in vivo* when mice are infected intravenously (15, 16). Thus, to specifically address if the early decrease in *Mtb* CFU burden in the mutant infection was responsible for increased iBALT formation, we infected mice with a 5-fold higher dose (500 CFU) of *Mtb* Δ*mmpl7* to directly compare the ability of Δ*mmpl7* mutant to induce iBALT when compared to low dose (100 CFU) infection with wildtype (WT) *Mtb* Erdman (Supplemental Figure 1). Using the higher dose model, our data show that Δ*mmpl7* mutant still had early defects in *Mtb* CFU *in vivo* in the lung and spleen as previously shown, but at 40 d.p.i. showed comparable CFU to WT *Mtb* and similar overall inflammation (Supplemental Figures 1A,B). Importantly, the higher dose model maintained the observed increased iBALT formation (Supplemental Figure 1C). Whether this phenotype was due partially or entirely to the lack of PDIMs on the MOM or due to other changes in the Δ*mmpl7* mutant was further evaluated in this study.

Furthermore, by 40 d.p.i., no differences in bacterial burden were observed in the lung or spleen between WT Erdman and Δ*mmpl7* mutant, which is more likely due to increased control of WT Erdman while Δ*mmpl7* mutant CFU levels were maintained. These findings demonstrate that our 5-fold higher CFU dose aerosol infection used to compensate for the growth defect in the Δ*mmpl7* mutant still recapitulates the *in vivo* phenotype observed with low dose infection. These data suggest that the observed phenotype of increased iBALT formation in the Δ*mmpl7* mutant infection is likely not an artifact of abrogated *Mtb* growth.

### Δ*mmpl7* Mutant *Mtb* Limits Early Myeloid Cell Accumulation in the *Mtb*-Infected Lung

**Figure 3:**
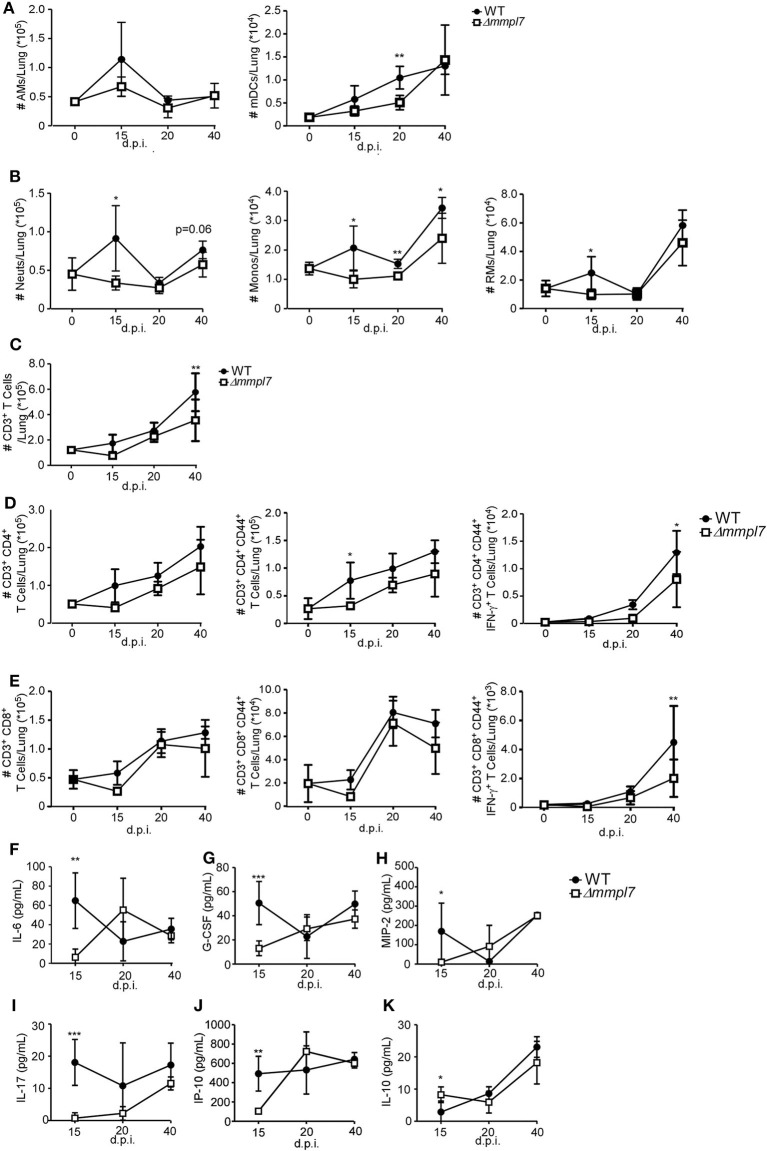
Δmmpl7 mutant Mtb limits myeloid cell accumulation in the lung: Sex and age matched C57BL/6 mice were infected with 100 CFU *Mtb* Erdman WT or 500 CFU Δ*mmpl7*. Single cell suspensions were processed from infected lungs and immune cell subsets were analyzed by flow cytometry. **(A)** tissue resident myeloid and **(B)** recruited myeloid cell accumulation in the *Mtb*-infected lungs was determined at indicated time points. **(C)** CD3^+^ lymphocytes, **(D)** CD4^+^ or **(E)** CD8^+^ T cells were determined using flow cytometry. **(F–K)** Protein levels of inflammatory cytokine and chemokines IL-6, G-CSF, MIP-2(CXCL2), IL-17, IP-10 (CXCL10), and IL-10 were determined in lung homogenates at indicated time points. Infected groups (*n* = 5) comparing WT Erdman and Δ*mmpl7 Mtb* strains at individual time points were compared using Student's *t*-test. Individual time points within the same group were compared using 2-way ANOVA with Bonferroni post-tests. Mean and standard deviation (SD) were plotted for each group at each indicated time point. *p < 0.05, **p < 0.01, ***p < 0.001.

To examine the early immune events that occur in the lung that mediate differences in iBALT formation, we infected mice with 100 CFU wildtype *Mtb* Erdman or 5-fold higher CFU (500 CFU) of the Δ*mmpl7* mutant and characterized myeloid and T cell accumulation in the lung. Upon infection with the Δ*mmpl7* mutant, we observed decreased neutrophils, monocytes and recruited macrophages at early time points as well as decreased accumulation of alveolar macrophages (AMs) and myeloid DCs (mDCs) when compared with accumulation in WT *Mtb* infected lungs (Figures 3A,B). We saw similar decreases in accumulation of total CD3^+^ T cells (Figure 3C), activated and IFN-γ producing CD4^+^ (Figure 3D) and CD8^+^ T cells (Figure 3E) at later time points.

These cellular changes coincided with early decreased production of inflammatory cytokines, such as IL-6, G-CSF, and IL-17, and chemokines, such as MIP-2 (CXCL2), and IP-10 (CXCL10) that are associated with myeloid recruitment in *Mtb* Δ*mmpl7* infected mice (Figures 3F–J). Interestingly, this coincided with an early increased induction of IL-10 at 15 d.p.i., though this effect was abolished by the later timepoints (Figure 3K). As IL-10 is a known mediator of anti-inflammatory responses (30, 31), these findings suggest a potential mechanism for the decrease in pro-inflammatory cytokines and lung inflammation. Taken together, these data demonstrate an overall “dampening” of the immune response during early infection with the Δ*mmpl7* mutant, suggesting that decreased inflammation and decreased neutrophilia may improve the formation of protective iBALT containing granulomas, thus imporving TB disease.

### Δ*mmpl*7 Mutant *Mtb* Overexpresses DATs and Limits Cytokine Production in Lung Epithelial Cells

**Figure 4:**
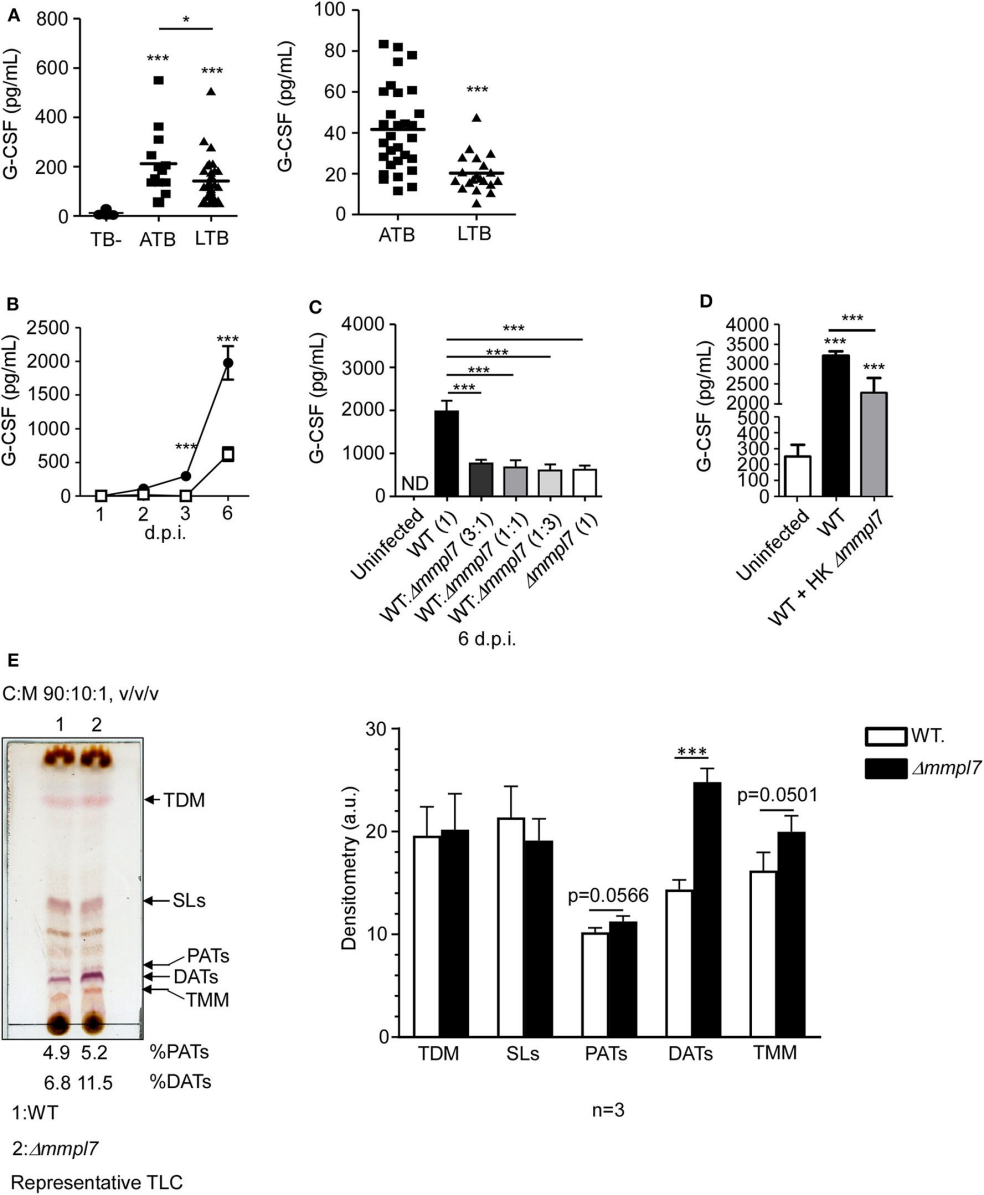
Δ*mmpl7* mutant Mtb overexpresses DATs and limits cytokine production in lung epithelial cells: **(A)** Serum samples from human active TB patients and controls (Left) Panel: Mexico (TB- *n* = 30; ATB *n* = 13; LTB *n* = 33), Right Panel: South Africa (ATB *n* = 30; LTB *n* = 20) were analyzed for G-CSF levels. Mouse lung epithelial cells (*n* ≥ 4 per group) were infected with *Mtb* Erdman WT or Δ*mmpl7* at **(B)** MOI = 1 or MOI = 5, respectively, for indicated time points. **(C)** Mouse lung epithelial cells (*n* = 4) were infected with *Mtb* Erdman WT and/or Δ*mmpl7* at indicated ratios for a constant total MOI = 1. **(D)** Mouse lung epithelial cells (*n* = 4) were infected with *Mtb* Erdman WT with or without the addition of heat-killed (HK) Δ*mmpl7*. **(A–D)** Supernatants were collected and G-CSF concentration was quantified by ELISA. **(E)** Total lipids from *Mtb* Erdman WT or Δ*mmpl7* were obtained from freshly grown bacteria (3 independent times) and extracted with chloroform:methanol (2:1, v/v/) after normalization by total bacterial number. Total lipids (100 μg) were analyzed by TLC using as a solvent system (chloroform:methanol:water, 90:10:1, v/v/v) and 10% sulfuric acid in ethanol as a developer to visualize total lipid contents (representative experiment shown). Densitometry analyses of the observed lipids were performed using the NIH software ImageJ, *n* = 3 (where each value corresponds to independent extractions from freshly grown bacteria and TLCs). Human cohort study groups were compared by 1-way ANOVA with Tukey's post-test, or unpaired Student's *t*-test, respectively. Infected groups comparing WT Erdman and Δ*mmpl7 Mtb* strains under indicated conditions at individual time points were compared using 1-way ANOVA with Tukey's post-test or unpaired Student's *t*-test. TLC densitometry was analyzed by unpaired Student's *t*-test. *p < 0.05, **p < 0.01, ***p < 0.001.

Having observed abrogated recruitment of myeloid cells after infection with the *Mtb* Δ*mmpl7* mutant and decreased cytokine/chemokine production *in vivo* (Figure 3), we hypothesized that the Δ*mmpl7* mutant was specifically modulating inflammatory molecule production and subsequent recruitment of immune cells. Increased early IL-10 production and decreased neutrophilia and inflammation observed after infection with the Δ*mmpl7* mutant likely skewed the immune response toward protective iBALT containing granulomas. Alternatively, increased neutrophil accumulation and increased pro-inflammatory cytokines, including G-CSF and IL-6, are induced in response to WT *Mtb* infection and drive non-protective, necrotic granulomas (10, 11, 32). To address if G-CSF was a marker of active TB disease in humans, we measured levels in individuals with active TB disease (ATB) or latent *Mtb* infection (LTBI), in two human cohorts. We observed that G-CSF production was increased in ATB individuals when compared with LTBI or house contacts (HCs) (Figure 4A). Furthermore, it was previously demonstrated that individuals with ATB also have increased serum levels of IL-6 (33, 34). As such, both G-CSF and IL-6 were used as cytokine indicators of inflammatory response severity for the remainder of this study.

To further test this hypothesis in our model, we examined mouse lung epithelial cells *in vitro*, which are a key source of G-CSF, and other inflammatory cytokines in the context of lung infection (5, 35–39). We assayed epithelial cell derived G-CSF production *in vitro* after infection with either wildtype *Mtb* Erdman or Δ*mmpl7* mutant. Firstly, we found that epithelial cells infected with WT *Mtb* induced significant G-CSF in a time-dependent manner. In contrast, infection of epithelial cells with Δ*mmpl7* mutant resulted in significantly decreased G-CSF production when compared to wildtype Erdman infection (Figure 4B). When performing coinfection with varying ratios of wildtype Erdman and Δ*mmpl7* mutant, we observed that even 3 times the presence of WT *Mtb* did not reverse the abrogated G-CSF production observed upon Δ*mmpl7* mutant infection (Figure 4C). Furthermore, we observed a significant decrease in G-CSF production even when wildtype Erdman infected epithelial cells were co-administered heat-killed (HK) Δ*mmpl7* mutant, suggesting that the inhibition of G-CSF was dependent on a factor expressed by *Mtb* Δ*mmpl7* and did not depend on the viability or infectivity of the bacteria (Figure 4D). These data together suggest that a molecular factor present in the Δ*mmpl7* mutant is likely limiting G-CSF expression *in vitro* and *in vivo*.

From these data, we hypothesized that a lack of *Mmpl7* in *Mtb* led to changes in the lipid profile of the MOM beyond the reported loss of PDIMs (15, 16, 40), thus inducing the presence of other lipids and virulence factors than those present on wildtype *Mtb* Erdman (41). We hypothesized that these lipids may decrease the production of G-CSF, IL-6 and other inflammatory cytokines, thus driving decreased myeloid recruitment and increased iBALT formation upon infection. To test this, we freshly grew *Mtb* WT and Δ*mmpl7* mutant bacteria three independent times, normalized by bacteria weight, and extracted and characterized their total lipid content by 1 and 2 dimensional thin layer chromatography (1D or 2D TLC) (Figure 4E and Supplemental Figure 2), using different solvents systems allowing us to fully screen the wide range of lipids present on the mycobacterial cell envelope. We found that while sulpholipids (SLs), trehalose dimycolates (TDMs) and trehalose monomycolates (TMMs) were present at similar levels, diacyl trehaloses (DATs) were expressed at higher levels in the cell envelope of the Δ*mmpl7* mutant *Mtb* (11.5% of total lipid) when compared to WT Erdman (6.8% of total lipid) (Figure 4E and Supplemental Figure 2, black arrows). DATs are heterogeneous glycolipids (>30 molecular species described) composed of a trehalose sugar structure with two acyl chains, where the trehalose moiety is thought to bind to the macrophage-inducible C-type lectin Mincle (42, 43). DATs have been previously shown to negatively regulate the pro-inflammatory response of the host, thus promoting *Mtb* survival (18, 19). Thus, these findings suggested that increased presence of DATs in the Δ*mmpl7* mutant could be linked to the decreased production of inflammatory molecules like G-CSF and IL-6 and the dampened pulmonary inflammatory response observed *in vitro* and *in vivo* (19).

### DAT Administration Drives Decreased Inflammatory Molecule Production in Epithelial and Myeloid Cells

**Figure 5:**
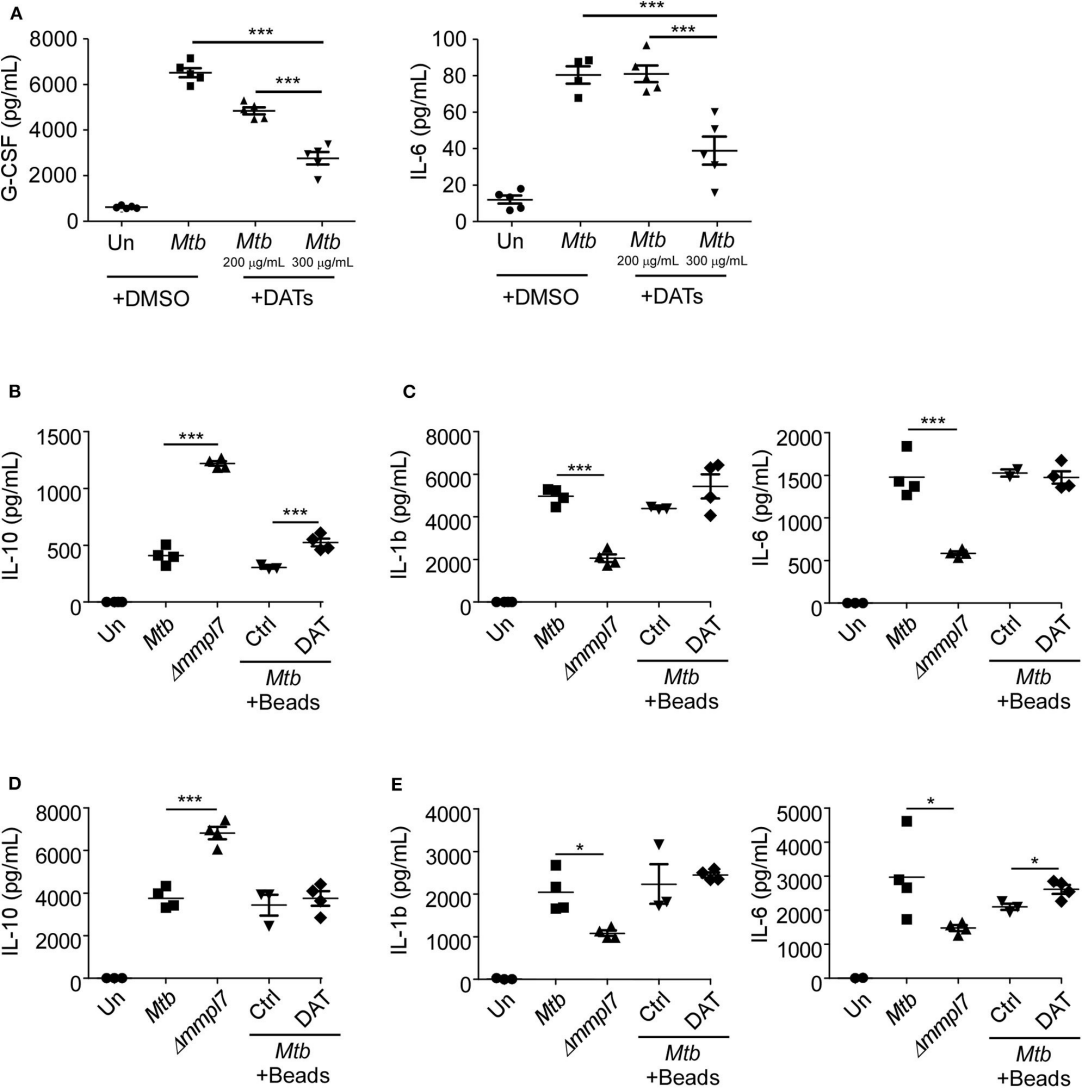
**(A)** DAT administration drives decreased inflammatory molecule production in epithelial and myeloid cells: Mouse lung epithelial cells (*n* = 5) were infected with *Mtb* Erdman WT with or without the addition of raw DATs at MOI = 1 for 6 days. Mouse bone-marrow derived **(B,C)** macrophages (BMDMs) (*n* = 5) or **(D,E)** dendritic cells (BMDCs) (*n* = 5) were infected with *Mtb* Erdman WT with or without the addition of DAT coated agarose beads, or Δ*mmpl7* for 3 days. Supernatants were collected and the concentrations of cytokines G-CSF, IL-6, IL-1β, and IL-10 were quantified by multiplex assays. Multiple groups were compared by 1-way ANOVA with Tukey's post-tests. *p < 0.05, **p < 0.01, ***p < 0.001.

We next hypothesized that the increased expression of DATs could abrogate production of cytokines, especially G-CSF, in epithelial cells (5, 7), similar to what we observed with infection with the Δ*mmpl7* mutant. To demonstrate this, we infected lung epithelial cells with *Mtb* Erdman and co-treated them with DATs. Our results show that co-treatment with DATs limited the production of G-CSF and IL-6 by WT *Mtb* infected epithelial cells (Figure 5A), similar to the responses observed when Δ*mmpl7* mutant single infection and coinfections were carried out (Figure 4). Additionally, the inhibitory effect observed was dose dependent, thus confirming that the overexpression of DATs was one factor that was skewing the induction of cytokines upon infection with the Δ*mmpl7* mutant. This effect was likely not due to DAT-induced cell death, as levels of total live, apoptotic, and dead cells were only slightly altered after addition of DATs (Supplemental Figure 3A). IL-10 production within this culture was below the limits of detection of the assay for all groups, even with addition of DATs, thus suggesting that epithelial cells are likely not a significant source of anti-inflammatory signals in this phenomenon.

We next aimed to determine if this phenotype was specific to epithelial cells or if the Δ*mmpl7* mutant also regulated cytokine expression in myeloid phagocytes, and if the presence of DATs modulated these responses as previously shown (19). Therefore, we infected macrophages and DCs with WT and Δ*mmpl7* mutant (Figures 5B–E). We found that while Δ*mmpl7* mutant abrogated IL-1β and IL-6 expression in both macrophages and DCs (Figures 5C,E), the Δ*mmpl7* mutant surprisingly induced increased IL-10 expression in both cell types when compared to WT *Mtb* (Figures 5B,D). Furthermore, no differences in cell death were observed between WT *Mtb* and the Δ*mmpl7* mutant (Supplemental Figures 3B,C), thus suggesting that this finding is due to specific regulation of cytokine production, not apoptosis induced abrogation. The decreased production of IL-1β and IL-6 in phagocytes was likely not only due to active inhibition by DAT alone, as co-treatment with DAT-coated beads did not decrease production of these cytokines during WT infection when compared to control beads. However, in BMDMs the increased induction of IL-10 driven by the Δ*mmpl7* mutant *Mtb* over WT Erdman was recapitulated by addition of DATs to WT Erdman infection (Figure 5B), thus suggesting that DAT overexpression on Δ*mmpl7* mutant *Mtb* actively induced expression of anti-inflammatory IL-10 produced by macrophages. The increased production of anti-inflammatory IL-10 by lung macrophages may explain the early increased IL-10 observed *in vivo* in lung homogenates. These early anti-inflammatory signals may abrogate the production of inflammatory cytokines and pulmonary neutrophilia, thus skewing the immune response away from neutrophil rich, non-protective granulomas, and toward iBALT containing, protective granulomas.

## Discussion

Although pulmonary *Mtb* infection is the leading cause of death by an infectious agent worldwide, the initial steps in pathogenesis that govern the induction of iBALT formation within granulomas remain unknown. In this study, using a transposon library in the NHP pulmonary infection model, we have identified *Mtb* drivers of B cell-containing iBALT formation. Our work here demonstrates that murine infection with the Δ*mmpl7* mutant induces increased iBALT formation by dampening early innate immune responses. The Δ*mmpl7* mutant overexpressed DATs in the cell wall, which drove abrogated inflammatory molecule production and increased IL-10 in the lung, thus leading to decreased cellular recruitment. This study provides novel evidence for a critical role for *Mtb* specific factors in skewing the earliest host pathogen interactions that drive protective or detrimental disease outcomes.

Although significant advances have been made in understanding the factors that aid in the intracellular survival of *Mtb* and their potential as therapeutic targets using *in vitro* approaches and the mouse model, it is unclear if these factors play important roles in human tubercle formation. Furthermore, most physiological events cannot be modeled by targeting a single gene or its substrates, as interaction and/or compensation among several genes/pathways underpin complex outcomes. Thus, in this study we have examined the *Mtb* determinants that mediate granulomas by using the well-established preclinical model of NHP TB infection. The macaque model replicates many facets of clinically observed *Mtb* infection, thus providing relevance to identification of *Mtb* determinants that drive or limit granuloma and iBALT formation. By infecting NHPs with an *Mtb* mutant transposon library we have identified a novel subset of *Mtb* genes associated with protective iBALT containing granulomas that correspond with effective *Mtb* control. Although the gene subsets identified using this approach did not fall into any specific pathway, they were interrelated in that they were predominantly involved in mycobacterial growth and survival via responses to stress (*Acr2, ClpB, Gln4, NdhA*, and *Cmtr*) or the synthesis/translocation of virulence components including lipids & proteins (*Mmpl2, Mmpl7, EccD5*).

Among the two heat stress molecular chaperones identified in our study *ClpB* has already been established as essential for the *in vitro* growth of *Mtb* (27), however the importance of *Acr2* is yet to be established. *Acr2* is the most up-regulated gene following phagocytosis of *Mtb* by macrophages (27, 44) and is under the control of the master regulator *PhoP* (45). Wilkinson et al. found that *Acr2* was strongly expressed in response to heat shock protein (HSP) *Rv0251c* and appears to play a role in early immune responses (46). HSPs assist in *Mtb* survival but also act as signaling agents to the host inflammatory mechanisms. While the role of *Acr2* in *Mtb* virulence in humans is not clear, it remains essential for virulence in a murine model of TB (46). To our knowledge this is the first study to report on the association of absence of *Acr2* with protective granulomas in the NHP model.

Until recently *NdhA* was considered a non-essential protein found in the inner mycobacterial membrane associated with nicotinamide adenine dinucleotide (NADH) mediated electron transfer to the electron transport chain (25). Recent studies by Vilcheze et al. concluded that when *NdhA* is the only type I NADH dehydrogenase present in *Mtb* it affects *Mtb* growth and renders it susceptible to oxidative stress (47). Likewise, while *GlnA2* and *GlnA4* are not associated with *Mtb* virulence *in vivo* another member of this family *GlnA1* was found to contribute to *Mtb* virulence in a guinea pig model (48). Also, while a direct role for *Cmtr* in *Mtb* virulence is yet to be established, a transcriptional survey of intracellular mycobacteria and their host macrophages revealed signatures of heavy metal poisoning and an associated strong induction of *Cmtr* and *Csor* which are known to encode metal responsive transcriptional regulators (49).

The *Mtb* cell wall expresses a variety of virulence factors that contribute to bacterial survival and intrinsic drug resistance. Identification of genes that regulate uptake and secretion machinery across the membrane is critical to characterizing the corresponding secretory products and thus the pathogenesis of *Mtb*. *EccD5* has been identified as being associated with protective granulomas. *EccD5* as part of the ESX-5 system is involved in the translocation of Proline-Proline Glutamate (PPE) proteins and its disruption affects cell wall integrity leading to strong attenuation of the pathogen in a mouse model (26). But the impact of *EccD5* in human *Mtb* infection or in NHPs is not known. Lastly, we also identified *Mmpl2* and *Mmpl7* to be associated with protective granulomas in the NHP model. Sequencing of the *Mtb* genome revealed 12 membrane proteins that were primarily involved in transport of *Mtb* lipids. *MmpL*-mediated lipid secretion impacts both the innate ability of the pathogen to survive intracellularly and also the host-pathogen interactions that determine the disease outcome. It has been previously established that only *Mmpl4* and the well-characterized *Mmpl7*, which transports PDIM to the MOM, have both impaired growth kinetics and impaired lethality (50). In summary, our *Mtb* mutant transposon library infection model in the NHPs has identified for the first time several unique *Mtb* genes whose roles in *Mtb* virulence were previously considered as redundant.

Since *Mmpl7* is the most well-characterized of all *Mmpl* genes being the known transporter of the virulence lipid PDIM, and as the substrate for *Mmpl2* is currently not known (51), we decided to focus our further studies utilizing the *Mmpl7* mutant strain (Δ*mmpl7*). Beyond reduced expression of PDIM, the Δ*mmpl7* mutant also has an established *in vivo* growth defect in the lung, spleen, and liver of mice, which we compensated for in our study by infecting with higher doses of the mutant compared to wild type. The decreased CFU in the lungs of Δ*mmpl7* mutant infected mice that we observed is similar to previous findings by Cox et al. and others (52), but the decreased dissemination to the spleen was not previously observed. This is likely due to the use of different background *Mtb* strains and routes of infection in our study. Furthermore, our higher dose aerosol infection recapitulates a similar phenotype in increased iBALT formation as the standard low dose aerosol infection. Thus, our findings suggest that the improved TB disease outcomes observed is likely not simply due to the absence of PDIMs or a growth defect of the mutant, but that the observed increased iBALT formation is likely driven specifically by the Δ*mmpl7* mutant and overpression of DATs.

Despite using a higher dose of the Δ*mmpl7* mutant in our infection studies, we observed enhanced iBALT formation and abrogated inflammatory molecule production and cellular recruitment, including both myeloid and lymphoid cell types. As T cells are required for the formation of iBALT, it would seem that slightly decreased T cell accumulation should also yield decreased iBALT, which was not the case. However, our main observed differences in cellular recruitment involved decreased early neutrophil associated responses, specifically abrogated cytokines like IL-6 and G-CSF, and increased IL-10. It is known that neutrophils are overrepresented in non-protective, necrotic granulomas that do not control or contain *Mtb*, and that neutrophil counts in peripheral blood, as well as IL-6 levels, correlate with ATB disease (10, 32, 33).

Our study confirms these findings with heightened blood levels of G-CSF in people with active TB when compared to LTBI, and as such we used these two inflammatory cytokines as readouts for response severity for our *in vitro* experiments. Thus, these data suggest that a lack of IL-6 and G-CSF and decreased neutrophils may be skewing toward a dampened immune response, potentially mediated by increased early IL-10, that allows for the enhanced formation of iBALT containing granulomas as opposed to necrotic, neutrophil containing lesions. This hypothesis is also supported by our findings *in vitro*, which show decreased IL-6 and G-CSF in lung epithelial cells, a key source of inflammatory molecules, after Δ*mmpl7* mutant infection.

Importantly, we observed increased IL-10 production in macrophages and DCs after Δ*mmpl7* mutant infection. It is known that IL-10 antagonizes IL-17 production, and thus downstream G-CSF production (53), suggesting a potential mechanism for our observed dampened immune responses and improved outcomes. Indeed, *Mtb* is capable of dampening immune responses mediated by pattern recognition receptors (PRRs), and not just stimulating heightened inflammatory responses by interactions with these receptors.

*MmpL7* is colocalized with genes involved in polyketide biosynthesis (pks genes) and genes involved in lipid metabolism (PapA, FadD) suggesting that it is involved in complex lipid transport involving more than one substrate in Mtb (54, 55). Therefore, we aimed to determine if there were any other differences in the lipid profile of *Mtb* beyond the loss of PDIM that could explain the observed abrogated cytokine responses *in vivo* and *in vitro*. We found that DATs are overrepresented in the Δ*mmpl7* mutant, validated by both 1D and 2D thin layer chromatography (TLC). *MmpL10* is considered to be the putative agent responsible for the transport of DAT across the plasma membrane (51). Hence, it is likely that the absence of *MmpL7* during infection could be compensated by *MmpL10* function accounting for the observed accumulation of DATs. The addition of DATs during WT *Mtb* infection recapitulated our findings of decreased inflammatory cytokine production in epithelial cells and yielded increased IL-10 production by macrophages, both supporting what was observed in Δ*mmpl7* mutant infection *in vivo*. These findings suggest distinct mechanisms for driving dampened immune responses depending on cell types, with likely interactions of IL-10 sourced from macrophages driving decreased production of inflammatory cytokines by epithelial cells. DATs likely do not act in isolation, as addition of DATs to WT *Mtb* Erdman infection did not fully recapitulate all aspects of Δ*mmpl7* mutant infection *in vitro* in these model systems.

Mycobacterial lipid virulence factors such as PDIMs are known to mask pathogen associated molecular patterns (PAMPs) and the resultant downstream PRR signaling (40). Lipid virulence factors are also known to directly act as toll-like receptor (TLR) antagonists (56). Apart from TLRs, other surface receptors including Mincle, that recognizes trehalose moieties (42, 43), are likely involved in this phenomenon as BMDMs from Mincle deficient mice reportedly produce less G-CSF and TNF in response to *Mtb* infection (57). Moreover, the expression of TLRs is also known to vary between different phagocytic cell types (58). Hence these separate responses are likely due to differences in PRR expression and signaling by these cell types. Interestingly, previous studies have shown DAT dependent abrogation of inflammatory cytokine production in monocytes and macrophages *in vitro* (19). This study was performed using several different human phagocyte models without abrogating the expression of Mincle. While Mincle is established to interact with trehalose moieties, this study validates our claim that DATs can drive abrogated inflammatory cytokine production, independent of any manipulation of the expression of Mincle, suggesting a separate mechanism. It is likely that the interactions and modulated cytokine/chemokine production we have observed involves other PRRs and *Mtb* lipid factors, beyond the singular interaction between DATs and Mincle (43).

While the various immune cells and cytokine/chemokine signals that contribute to iBALT formation are well-established in a variety of disease contexts (59), the *Mtb* specific factors that drive these protective responses, and the mechanism of those interactions, are poorly understood. This study demonstrates that a single *Mtb* specific lipid factor can differentially affect the ability of various cell types to produce inflammatory and anti-inflammatory cytokines, all of which contribute in different aspects to the milieu that fosters conditions beneficial to iBALT formation. In the context of an immune structure as complex as iBALT, where multiple coordinated and regulated signals and cell types are required, future work will likely need to use a similar approach across several *in vivo* and *in vitro* model systems examining multiple cell types in order to elucidate the concise contribution of a specific *Mtb* factor and PRR interactions. Future work may also need to examine not only multiple cell types, but also locational and temporal cellular interactions throughout the course of infection, as a single *Mtb* factor is likely neither necessary nor sufficient in isolation to drive iBALT by interactions with a single cell type at one specific time point.

While this study was not exhaustive in analyzing all potential lipid factors involved, it provides a framework for determining the contributions of the other *Mtb* genes and potential candidate lipids in skewing toward protective outcomes. We acknowledge that the role of any specific lipid in skewing iBALT formation is not an isolated phenomenon, as *Mtb* mutants knocking out certain lipids inevitably drive compensatory overexpression of others. Furthermore, we expect that the increased iBALT formation we have observed is likely due to a combination of a lack of PDIM along with increased DATs, with the possibility of other lipids being involved that we did not identify or assay for.

Our novel findings demonstrate that genes associated with *Mtb* cell wall lipids are critical to the initial interactions between *Mtb* and the host and suggest that *Mtb* specific lipids are key determinants of the early immune response that skews toward formation of iBALT. Our findings further provide a list of *Mtb* gene candidates for future work examining conditions needed for protective iBALT formation, specifically aimed at early responses that determine these outcomes. We have also demonstrated for the first time a panel of novel *Mtb* genes that are associated with *enhanced formation of protective iBALT containing granulomas and improved disease outcomes*, while most work on this topic examines genes involved in detrimental host outcomes and loss of protection. This study thus provides a framework for future attenuated vaccine candidates and mechanistic studies across model systems.

## Materials and Methods

### Non-human Primate Infection

An *Mtb* (H37Rv) transposon site hybridization mutants (TraSH) library (kind gift of Dr. Chris Sassetti) was delivered high dose (≥100,000) CFU via bronchoscopic procedure into the lungs of triplicate Indian rhesus macaques. Delivery of bacteria to the lower lungs was confirmed via dilution plating and *Mtb* infection was confirmed by direct observation of the clinical signs of TB and concurrent positive tuberculin skin test. NHPs were humanely euthanized after being held 4–6 weeks due to development of disease using prespecified criteria (60–62). Lungs from infected animals were harvested and processed for mesodissection as previously described (63, 64). Briefly, lung sections were removed, formalin fixed and paraffin embedded. A series of unstained and H&E slides were then made and used with the mesodissection instrument and 2iD Imaging software. The H&E guide slide is used to direct the consumable mill bit to dissect the unstained slide and harvest the nuclear material for downstream upscaling and identification. By staining of B cells, iBALT associated lesions were first identified. The appropriate sections from H&E slides were then dissected and used for sequencing as previously described (65) to identify the single *Mtb* mutant associated with each individual lesion (23, 24).

### Mice

C57Bl/6 (B6) mice used were 6–8 weeks old and sex matched. All treatments and conditions used to handle study animals are in accordance with the approved Institutional Animal Care and Use Committee (IACUC) guidelines at Washington University in St. Louis.

### Bacterial Strains, Infection and Instillation

*M*tb (Erdman) and Δ*mmpL7* mutant (MJM39) (16) were stocked in Proskauer-Beck with 0.05% tween 80 and stored at −80°C. Prior to infection, frozen stocks were thawed and placed in a solution of sterile PBS for loading into the Glasscol nebulizer. Mice were infected with aerosolized *Mtb* in the above Glasscol nebulizer at tested doses of ~100 CFU (low) or ~500 CFU (5-fold higher) of *Mtb* per mouse. At the indicated time points mice were sacrificed via carbon dioxide narcosis and lungs and spleens were harvested.

### Microscopy and Inflammation, B Cell Quantification

Lung pathology from formalin fixed paraffin embedded lungs was assessed digitally using the automated tool of the Zeiss Axioplan 2 microscope (Carl Zeiss) of H&E stained slides to quantify inflammation and immunofluorescence labeled slides B220, CD3 and counter stained with DAPI in order to assess lymphoid follicle area.

### Bacterial Culture and Cytokine Analysis

Bacterial burden was assessed using serial 10-fold dilutions of lung or spleen and plated on 7H11 supplemented with OADC (oleic acid, bovine albumin, dextrose, and catalase). After 2–3 weeks incubation colonies were counted visually. Cytokine/chemokine expression was analyzed in lung homogenates from infected mice via Luminex (Millipore-Sigma) or ELISA (R&D).

### Flow Cytometry

Flow cytometry was conducted on single cell preparations derived from infected and uninfected lungs using fluorochrome conjugated antibodies:

Myeloid antigen presenting cell panel (66): CD11b (M1/70), CD11c (HL3), Gr-1 (RB6-8C5), Siglec-F (E50-2440), Ly6G (1A8), Ly6C (AL-21), CD64 (X54-5/7.1), MHC-II (M5/114.15.2). Cells were defined as: AMs: CD11b^−^, CD11c^+^, Siglec-F+, CD64+. mDCs: CD11b^+^CD11c^+^, MHC-II^+^. Neutrophils: CD11b^+^, CD11c^−^, Gr-1^hi^. Monocytes: CD11b^+^ CD11c^−^ Gr-1^int^ and/or Ly6C^+^. Recruited macrophages: CD11b^+^ CD11c^−^, Gr-1^lo^, CD64^+/−^, Ly6C^+/−^, MHC-II^+/−^.

Activated lymphocyte panel (9, 67): CD3ε (500A2), CD4 (RM4-5), CB8α (53-6.7), CD44 (IM7), IFN-γ (XMG1.2).

Live, apoptotic, or dead cell panel (66): Annexin V (PE) and 7-AAD (PerCP-Cy5.5) from the BD Pharmingen Apoptosis Detection kit were utilized and cell percentages were quantified by flow cytometry as per manufacturer's suggested protocol (BD Biosciences). The percentage of cells were defined as live (Annexin V^−^, 7-AAD^−^), apoptotic (Annexin V^+^, 7-AAD^−^), and dead (Annexin V^+^, 7-AAD^+^).

Data collection and analysis were conducted on the BD LSR Fortessa, Fortessa-X20 Cytometers or the FACSJazz Cell Sorter, all with FACS Diva software and post-acquisition analysis conducted on FlowJo.

### Human Serum Collection and Analysis

Human samples were collected on approval from the Ethics Committee of the National Institute for Respiratory Diseases, KwaZulu-Natal Research Institute for TB and HIV, Durban, South Africa; and The American British Cowdray Medical Center, Mexico City, Mexico on a protocol approved by the Ethics Committee of the National Institute for Respiratory Diseases (INER), KwaZulu-Natal Research Institute for TB and HIV (Currently renamed AHRI), South Africa and The American British Cowdray Medical Center. All subjects were of similar socioeconomic status and unrelated to the third generation as determined by a questionnaire. TB cases had symptoms (weight loss >10 kg, cough, fever, night sweats for >1 month, or cervical or axillary lymphadenopathy) and chest radiographic findings consistent with recent pulmonary TB, a positive sputum acid-fast smear and culture confirmed for *Mtb*. Fresh blood samples from active pulmonary TB patients were obtained from patients recruited to the Tuberculosis Outpatient Clinic, INER, Mexico, or the KwaZulu-Natal Research Institute for TB and HIV, South Africa. Serum samples from all patients with active TB were collected prior to anti-*Mtb* treatment and did not presented comorbidities such as diabetes, HIV, cancer, and COPD. LTBI patients were defined as asymptomatic individuals who had positive IGRA test results as previously described (10). We simultaneously recruited a group of HCs and these individuals were negative for IGRA tests at collection sites.

### *In vitro* Culture, Isolation, Stimulation, and Infection

Mouse lung epithelial cell line C10 cells were cultured to confluence in 24 well plates in DMEM. Prior to *in vitro* infection hemocytometer counts from a representative well were used to calculate multiplicity of infection (MOI). C10 cells were washed once with sterile PBS and then infected with *Mtb* or *Mtb* mutant at an MOI of 1 in DMEM without antibiotics and incubated at 37°C, 7.5% CO_2_. Supernatants from the infection was collected and stored at −80°C. Cells were collected by tryspinization and stained as per manufacturer's protocol.

Primary B6 mouse bone marrow derived macrophages (BMDMs) and dendritic cells (BMDC) were prepared as previously described (66). Briefly, bone marrow was harvested in a solution of serum free DMEM and then passed through a 70 μm screen, spun down and then resuspended in red blood cell lysis solution. Equal volume of cDMEM was added and again spun down. The single cell suspension was plated at 1 × 10^6^ cells per ml and supplemented with 4% GM-CSF for 7 days. At harvest, adherent cells were collected as BMDMs, and floating cells were collected as BMDCs, gently washed with CDMEM via centrifugation (1,200 rpm, 6 min at 4°C), resuspended and then plated at 1 × 10^6^ cells per ml.

Indicated cell types were stimulated with either DATs resuspended in DMSO, or BSA- control or DAT-coated beads suspended in PBS as indicated at 200 μg/mL. Briefly, beads (1.5 × 10^9^ Polybead polystyrene beads) were washed twice in 0.05 M carbonate-bicarbonate buffer (pH 9.6) and then incubated with 50 μg of DATs or buffer alone for 1 h at 37°C. Beads were then blocked with 5% BSA, washed repeatedly in 0.5% BSA, and finally adjusted to 4.0 × 10^8^/ml in 0.5% BSA before being used (68).

### Lipid Extraction and Analysis

*Mtb* bacterial pellets normalized by bacterial numbers (counted by microscopy, 1 × 10^10^) were extracted with chloroform:methanol (2:1, v/v) following the previously published method (17). Dried total lipids extracts were analyzed (loaded 100 μg by weight) by thin layer chromatography using chloroform:methanol:water (90:10:1, v/v/v) as the mobile phase, and 10% sulfuric acid in ethanol as a developer as described (68). Densitometry using NIH ImageJ software was used to quantify all the spots/lipids per lane in each TLC, and to calculate the percentage of total DATs and total polyacyl trehaloses (PATs) per lane. Densitometry analyses of each spot/lipids were also calculated as using NIH ImageJ software (*n* = 3, where each value corresponds to independent extractions and TLCs). DATs were purified by preparative TLC as previously described for other *Mtb* cell envelope lipids (68), and used for *in vitro* studies at the indicated concentrations, resuspended in DMSO.

### Statistical Analysis

Data analysis was conducted in GraphPad Prism 5 (La Jolla, CA) using unpaired two tailed Student's *t*-test for comparison between two groups or one-way analysis of variance for multiple comparisons. Significance is denoted as: ^*^p < 0.05, ^**^p < 0.01, ^***^p < 0.001, not detected, ND.

## Data Availability Statement

All datasets generated for this study are included in the article/Supplementary Material.

## Ethics Statement

The studies involving human participants were reviewed and approved by AHRI and INER ethics board. The patients/participants provided their written informed consent to participate in this study. The animal study was reviewed and approved by Washington University in St. Louis, Tulane Primate Center.

## Author Contributions

MD and OP designed and performed experiments, analyzed data, and prepared the manuscript. KT, JR-M, JS, and JT performed experiments, provided samples, and generated data. JC, JZ, and AS provided samples or reagents. DK and SK designed experiments and provided guidance and funding. All authors contributed to the article and approved the submitted version.

## Conflict of Interest

The authors declare that the research was conducted in the absence of any commercial or financial relationships that could be construed as a potential conflict of interest.
