# Supplementary figures and images for: Formation of Lung Inducible Bronchus Associated Lymphoid Tissue Is Regulated by Mycobacterium tuberculosis Expressed Determinants
Source: Front Immunol. 2020 Jun 30;11:1325. doi: 10.3389/fimmu.2020.01325 (PMC7338767; doi:10.3389/fimmu.2020.01325)

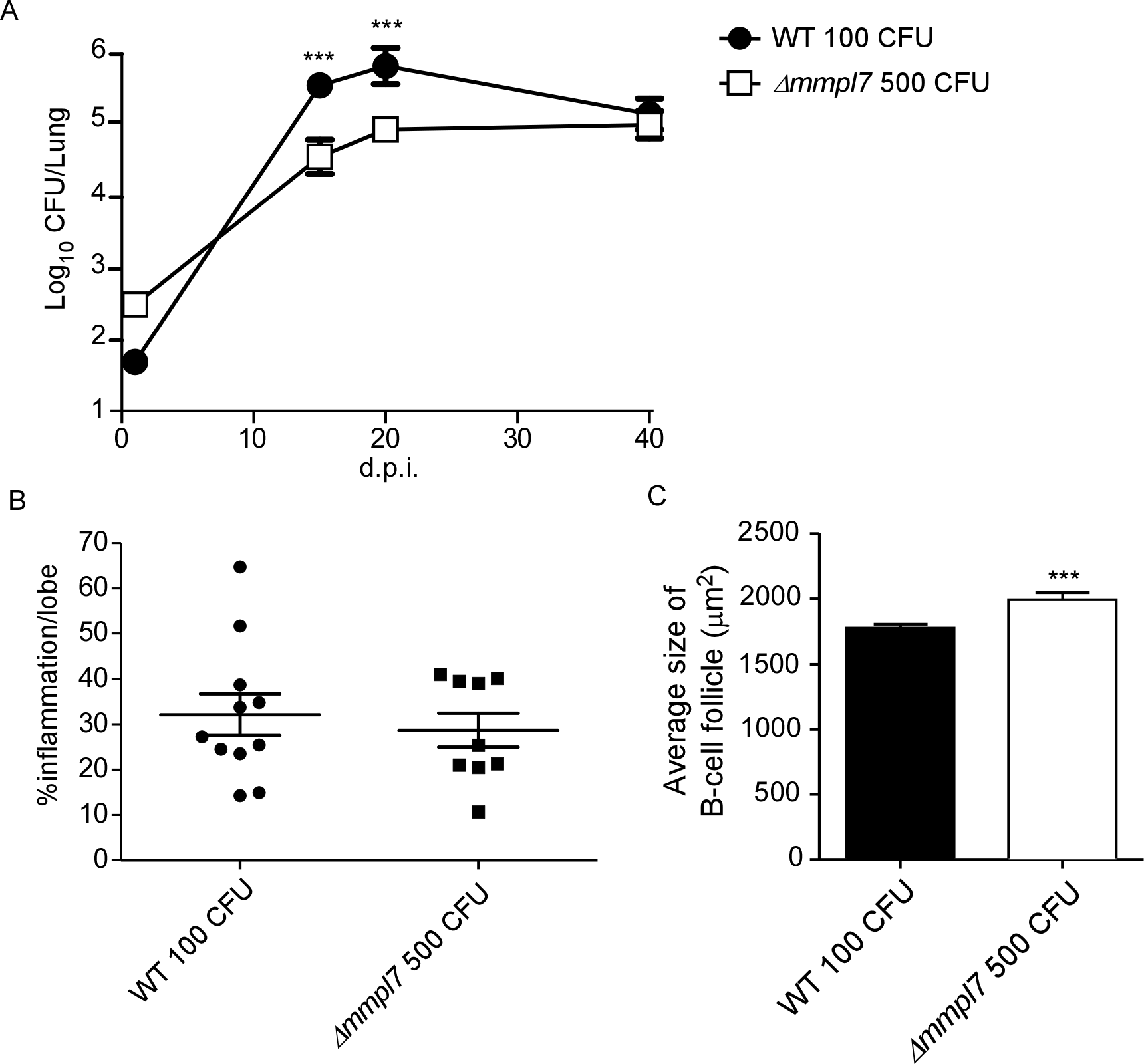

Supplement: Supplementary file 1 [file Image_1.TIF]

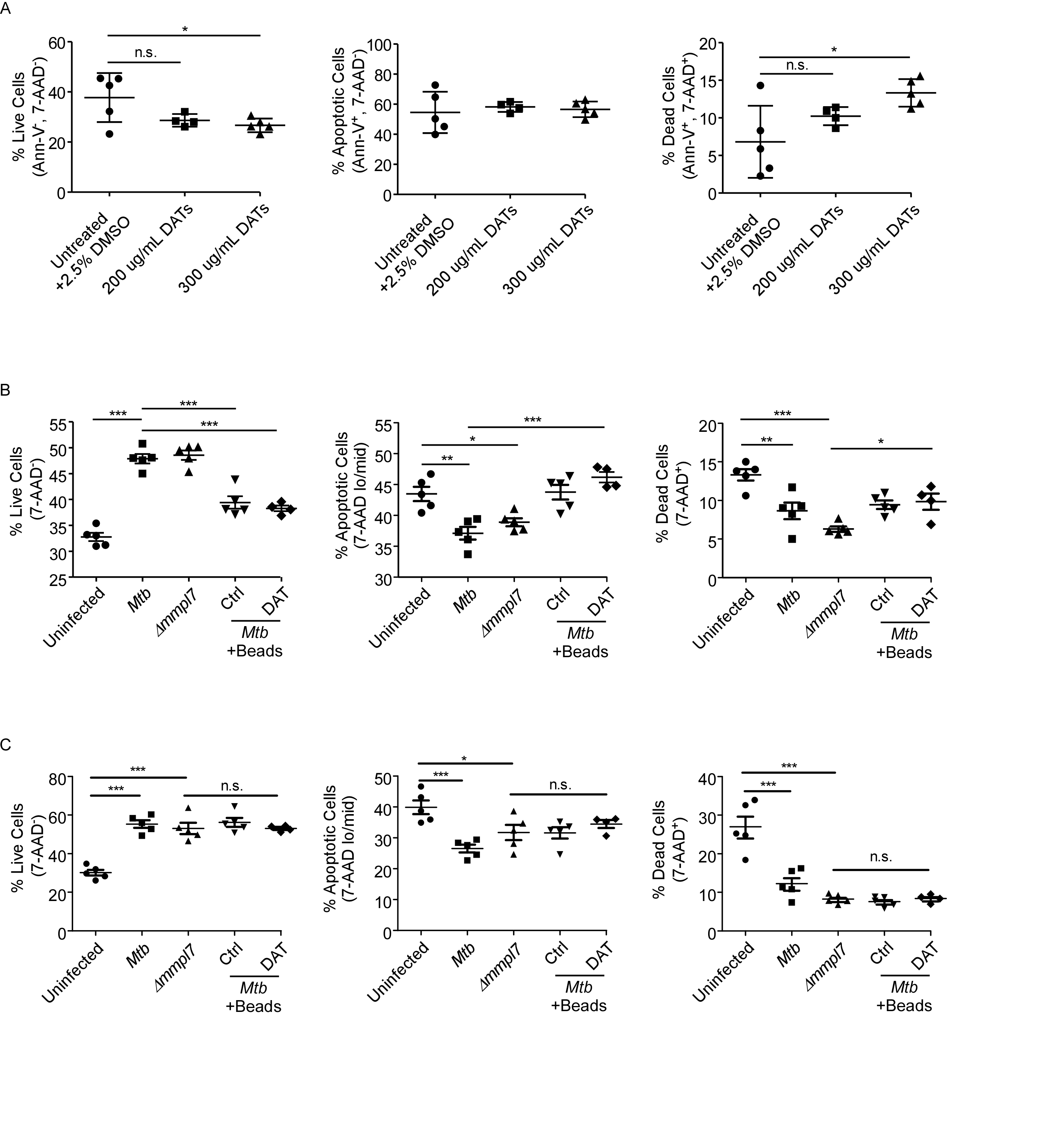

Supplement: Supplementary file 2 [file Image_2.TIF]

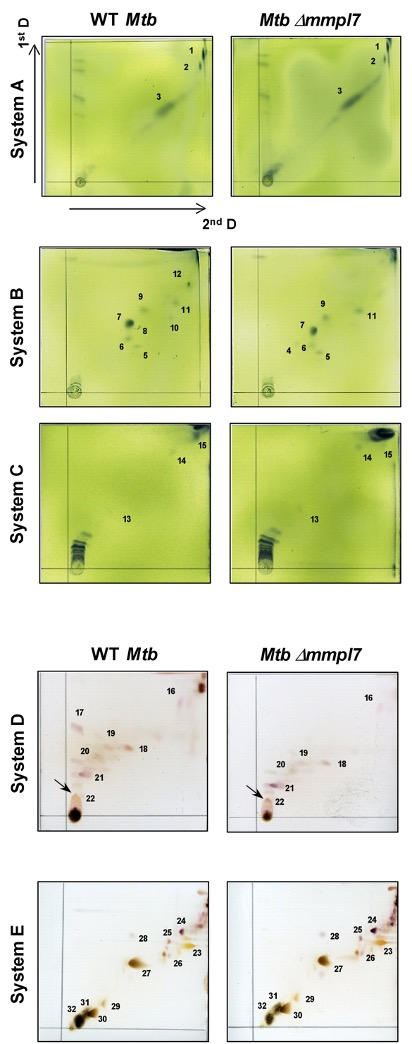

Supplement: Supplementary file 3 [file Image_3.JPEG]
