# Supplementary material for: Formation of Lung Inducible Bronchus Associated Lymphoid Tissue Is Regulated by Mycobacterium tuberculosis Expressed Determinants
Source: Front Immunol. 2020 Jun 30;11:1325. doi: 10.3389/fimmu.2020.01325 (PMC7338767; doi:10.3389/fimmu.2020.01325)
Supplement: Supplementary file 4 [file Data_Sheet_1.docx]

**Supplemental Figure 1: *Δmmpl7* *mutant drives Mtb enhanced B cell follicle formation in mouse model.***

Sex and age matched C57BL/6 mice were infected with 100 CFU of *Mtb* Erdman WT or 500 CFU *Δmmpl7*. A) Lung homogenates were taken and serially diluted to determine bacterial CFU counts at indicated time points. B) Lungs from *Mtb*-infected mice at 40 d.p.i. were formalin fixed, embedded in paraffin and used for H&E staining and inflammation was quantified by tracing areas. C) B-cell follicles present within lung sections were visualized by confocal microscopy. Slides were visualized and quantified by outlining the lesions using the automated tool of the Zeiss Axioplan 2 microscope. Infected groups (n≥5) comparing WT Erdman and *Δmmpl7* *Mtb* strains at individual time points were compared using Student’s t-test. Mean and standard deviation (SD) were plotted for each group at each indicated time point. *p<0.05, **p<0.01, ***p<0.001

**Supplemental Figure 2: *Δmmpl7 mutant Mtb overexpresses DATs.***

Two dimensional Thin Layer Chromatography was performed on total lipids from *Mtb* Erdman WT or *Δmmpl7* obtained as described in the main text and as resolved using the following solvent systems:

System A: (1) petroleum ether (boiling point 60–80 °C)/ethyl acetate (98 : 2, three times); (2) petroleum ether/acetone (98 : 2).

System B: (1) petroleum ether/acetone (92 : 8, three times); (2) toluene/acetone (95 : 5).

System C: (1^st^ D) chloroform/methanol (96 : 4); (2^nd^ D) toluene/acetone (80 : 20).

System D: (1) chloroform/methanol/water (100 : 14 : 0.8); (2) chloroform/acetone/methanol/water (50 : 60 : 2.5 : 3).

System E: (1) chloroform/methanol/water (60 : 30 : 6); (2) chloroform/acetic acid/methanol/water (40 : 25 : 3 : 6). Identities of separated lipid moieties 1-2: Phthiocerol dimycocerosate (PDIM) family; 3: Triacylglycerides (TAG); 4-8: Mycolipenates of Trehalose; 9: Free fatty acid (FA); 10-12: Unknown (?); 13: Phenolic Glycolipid-I (PGL-I); 14-15: Mixture of Free Fatty Acids (FA) and Diacyl glycerols (DAGs)*; 16: Sulfolipid-I (SL-I); 17: Unknown (?)**; 18: Glycolipid; 19-20: Sulfolipids (SL); 21: Trehalose Dimycolate (TDM); 22: Mixture of Diacyl trehaloses (DATs, black arrows) and Trehalose Monomycolate (MMT); 23: Diphosphatidyl-Glycerol (DPG); 24: Ac_2_PIM_2_; 25-26: Phospholipids (PC, PE, PS); 27: Ac_1_PIM_2_; 28: PI; 29: Ac_2_PIM_6_; 30: Ac_1_PIM_6_; 31-32: Lipooligosaccharides (LOSs).

**Supplemental Figure 3: *DAT administration does not significantly alter apoptosis and cell death in epithelial cells, macrophages, or DCs.***

A) Mouse lung epithelial cells (n=5) were treated with either 2.5% DMSO or raw DATs in DMSO at indicated dosage for 6 days. B) Mouse bone marrow derived Macrophages (BMDMs) (n=5) or C) dendritic cells (BMDCs) (n=5) were infected with *Mtb* Erdman WT with or without the addition of BSA control or DAT coated agarose beads, or *Δmmpl7* for 3 days. The percentage of live (left panels; Annexin V^-^, 7-AAD^-^), apoptotic (middle panels; Annexin V^+^, 7-AAD^-^), and dead (right panels; Annexin V^+^, 7-AAD^+^) cells were quantified by flow cytometry utilizing Annexin V and 7-AAD staining as per manufacturer’s suggested protocol. Multiple groups were compared by 1-way ANOVA with Tukey’s post-tests. *p<0.05, **p<0.01, ***p<0.001. Mean and standard deviation (SD) were plotted for each group at each indicated time point.
